# Food industry perspectives on potential policies targeting unhealthy food and beverage price promotions in Australian supermarkets

**DOI:** 10.1186/s12889-022-13812-7

**Published:** 2022-07-26

**Authors:** Lily Grigsby-Duffy, Adrian J Cameron, Kathryn Backholer, Gary Sacks

**Affiliations:** Global Obesity Centre (GLOBE), Institute for Health Transformation, Deakin University, Geelong, VIC 3220 Australia

**Keywords:** Nutrition policy, Price promotion, Marketing, Food environment, Stakeholder perspectives

## Abstract

Current supermarket price promotions are likely to encourage unhealthy diets, leading some governments to recently endorse restrictions on price promotions for unhealthy food and beverages. However, little is known about the likely industry response to policy action in this area. The aim of this study was to understand how potential government policies targeting food and beverage price promotions in supermarkets are perceived by food industry stakeholders in Australia. Twelve semi-structured in-depth interviews were conducted with current and former employees of major food manufacturers and food retailers as well as other industry experts with experience related to price promotion practices in the Australian supermarket setting. Data were analysed deductively based on Lewin’s organisational change theories and inductively to highlight forces that might drive or restrain change.

From an industry perspective, forces likely to create industry opposition to implementation of price promotion policy included: fear of losing competitive advantage; potential financial loss for food retailers and their suppliers; a perception that restrictions on price promotions for unhealthy products will not impact health; and a perception of increased financial cost to consumers. Forces perceived to drive implementation of a policy that would benefit public health included: mandatory regulation; extensive compliance monitoring; support for promoting healthy products; consumer education; and sufficient lead time and support from retailers for implementation. These forces, and the way in which they interact, need to be actively considered as part of efforts to change the healthiness of food and beverage price promotions in supermarkets.

**Supplementary Information:**

The online version contains supplementary material available at 10.1186/s12889-022-13812-7.

## Introduction

Supermarkets are the main source of food and beverage purchases in many countries [1–4]. Through the use of various marketing techniques that manipulate price, placement, promotion, and product (referred to as the ‘4Ps of marketing’), supermarkets have the ability to influence people’s purchasing and consumption behaviour [5]. As governments around the world seek to improve the healthiness of population diets [6], supermarkets are therefore an important focus area for policy action.

Price promotions are a common and effective marketing technique used in supermarkets to increase purchases [7], and are likely to increase the consumption of food and beverages bought on promotion [8]. A systematic review on the healthiness of food and beverage promotions published in 2020 found that seven out of eight studies observed a higher prevalence of unhealthy, compared to healthy, price promotions in food retail outlets [9]. A food environment that encourages the purchase of unhealthy foods and beverages is likely to contribute to the purchase and consumption of unhealthy diets.

Government policies aimed at changing the relative price of food and beverage so that unhealthy options are less financially attractive have been consistently recognised as an important part of efforts to create healthy food environments and improve population health [10–14]. For example, many countries have had success in reducing the purchase and/or consumption of unhealthy products after applying taxes to unhealthy foods or beverages [15–18]. However, in considering food prices and their influence on population health, the role of price promotions on unhealthy food and beverages warrants closer attention [19].

While there have not been any ‘real-world’ studies that have investigated the public health impact of reducing price promotions on unhealthy food and beverages in supermarkets, one recent modelling study assessed the potential cost-effectiveness of a policy restricting price promotions on sugar-sweetened beverages in Australian food retail settings [20]. That study estimated the policy was likely to reduce mean population per capita daily sugar intake, and would likely result in a reduction of mean population body weight [20], although the authors acknowledged the limited evidence for the real-world effectiveness, feasibility, and acceptability of such policy action.

In 2020, the UK Government announced legislation to restrict large and medium retailers that sell food and drink in-store and online from offering volume-based price promotions for foods and beverages high in fat, sugar, or salt [21]. Despite support from non-government organisations, charities, and public health bodies [22], the policy has received criticism from food industry stakeholders. Similar to industry’s response to other government-led food regulations, key criticisms cited by the UK food industry have included the potential loss of jobs as a result of the policy; a potential increase in overall food cost for consumers; and an expectation of only a modest impact on health due to the policy [23–25]. There was also concern expressed by some food industry stakeholders that a ban on promotions would be incompatible with other reformulation initiatives set by the government [23–25].

Outside of the UK, little is known about the food industry’s perceptions of government-led action to restrict unhealthy food and beverage price promotions and the potential impact that could have on the implementation and effectiveness of government policies in this area. Several previous studies have explored food industry attitudes towards a range of healthy retail marketing strategies and identified their reasons for marketing ‘less healthy’ foods [26–31]. Key factors included consumer demand for unhealthy foods, difficulties with storing some healthier foods, the higher cost of healthier foods relative to ‘less healthy’ foods, and incentives from manufacturers that maintain the status quo [26–31]. Much of the research in this area has focused on small, independent grocery stores [28–31], with participants that represent only the retailer side (without including the perspectives of food manufacturers) [28–31], and have not specifically focused on price promotions [26–31].

Exploring industry perspectives on government adoption and industry implementation of potential policy options can help provide an understanding of the context in which industry operates [32]; the power and resources available to them [33]; the likely impacts of policy action across the food system [10]; and potential opposition from food industry stakeholders that may be a barrier to policy implementation [10]. Furthermore, it is important to understand both food retailer and manufacturer perspectives given their joint influence on marketing decisions [26, 33]. Therefore, the aim of this study was to gather in-depth qualitative data to better understand current price promotion practices and factors that may hinder or help implementation of a policy to improve the healthiness of food and beverage price promotions in Australian supermarkets. Specifically, this study sought perspectives from food manufacturer and supermarket stakeholders involved in setting price promotions regarding: (a) the process by which price promotions are set; (b) the acceptability of potential policy actions that modify food and beverage price promotions to encourage healthy eating; and (c) the perceived feasibility, barriers, and facilitators to implementing policy actions that target food and beverage price promotions.

## Materials and methods

### Study design and theoretical framework

In-depth semi-structured interviews were conducted with a range of people with expertise on price promotions based on their first-hand experience working with Australian (1) food manufacturing companies and/or (2) supermarkets.

This analytical framework for the study drew on Lewin’s theories of organisational change. Lewin was a prominent researcher in the field of social psychology and developed several theories, to be used in conjunction with one another, to facilitate organisational change [34]. Lewin’s theories of organisational change have been widely applied in a range of fields including health promotion [35], health care [36–38], and business management [39, 40]. According to Lewin’s theories, before making change it is important to understand and map the ‘field’ (the group/organisational environment) and the ‘forces’ (driving and restraining) that influence the status quo [41].

Ethical approval was granted by the Deakin University Human Ethics Advisory Group (HEAG-H 131_2020). Participants received and returned a signed consent form prior to the interview. Additional verbal consent to audio-record the interviews was gained prior to each interview.

### Sample selection and recruitment

People with influence, experience and/or in-depth knowledge of the Australian supermarket setting and price promotion practices were purposively sampled. In the first instance, a range of current employees (from major supermarket chains and consultancy firms) known to the authors were contacted. The majority declined to participate, citing concerns around Australian regulations prohibiting collusion on price setting. Next, additional employees and ex-employees of relevant organisations were identified and invited to participate based on known contacts, Google searches, and the social media platform, LinkedIn. LinkedIn was searched by entering a relevant job title e.g., “promotions manager” into the search function and filtering by country (i.e., Australia) and industry (e.g., food and beverage). Snowball searching was used to find similar contacts (primarily using the function “People also viewed” on the LinkedIn platform). Additionally, key food and beverage company LinkedIn profiles were searched to identify employees.

Seven current and former employees of food and beverage retailers, suppliers and manufacturers in Australia were identified through LinkedIn. Three people known to the research team who have experience in Australian supermarket settings and price promotions were also recruited. An additional two participants were identified through snowball sampling. Recruitment was ceased once no new themes emerged and data saturation was reached (*n* = 12) [42].

### Data collection

Semi-structured in-depth interviews were conducted with the aid of a pre-developed interview guide. The interview guide was based on concepts from Lewin’s organisational change theories [41]. Key concepts from Lewin’s theories that were incorporated into the interview guide included: the current processes of the organisation (including operating procedures, the way decisions are made, and relevant stakeholders involved); identifying the perceived need for change and/or what it would take to trigger a perceived need; the ‘forces’ (internal or external to the organisation) that influence decision making; which ‘forces’ might help drive change and which might restrain change (including understanding any concerns); and identifying a variety of solutions to the perceived restraining forces [34, 41] (see Additional file 1).

Due to restrictions related to the Covid-19 pandemic, interviews were conducted on a one-to-one basis via the video calling platform Zoom. Interviews took an average time of 44 min (range: 33–59 min). Interviews were conducted by LGD over a six-month period (September 2020 - February 2021).

### Analysis

All interviews were recorded and transcribed. Transcripts were emailed to interviewees for review and to offer the opportunity to remove content deemed commercially sensitive.

Transcripts were imported into NVivo 12 for data analysis. Coding of themes was completed in two stages: a deductive approach using preliminary codes based on Lewin’s theories and previous research, followed by an inductive approach where codes were added or removed. Utilising a constant comparative method, analysis was performed alongside recruitment and data collection in order to refine and focus future interviews and to monitor data saturation [42]. Codes were organised into categories and sub-categories and iteratively reviewed to identify themes. A randomly selected transcript was coded by a second researcher to check agreement between coding. The overall coding of data and themes was reviewed by one of the research team (GS).

Themes were synthesised narratively and conceptualised into a Force Field Analysis Model, a visual representation of the identified forces restraining from, and driving towards, change. In this case, the desired change is healthier price promotion practices in Australian supermarkets. According to Lewin, plotting the driving and restraining ‘forces’ can facilitate the process of change by helping stakeholders to understand the ‘forces’ that require strengthening and/or reducing [34].

## Results

### Overview

Twelve stakeholders with experience and/or in-depth knowledge of food and/or beverage price promotion practices in the Australian supermarket setting were interviewed. The final sample included individuals with experience working for or consulting to food and beverage manufacturers (*n* = 5) and food retailers (*n* = 1), whilst the others (*n* = 6) had worked or consulted to both sectors. Three out of the 12 interviewees no longer directly worked in the food retail industry. Years of industry experience ranged from 4 years to over 20 years, with all individuals having had relevant managerial roles (including responsibility for relevant decision-making and team management).

**Fig. 1:**
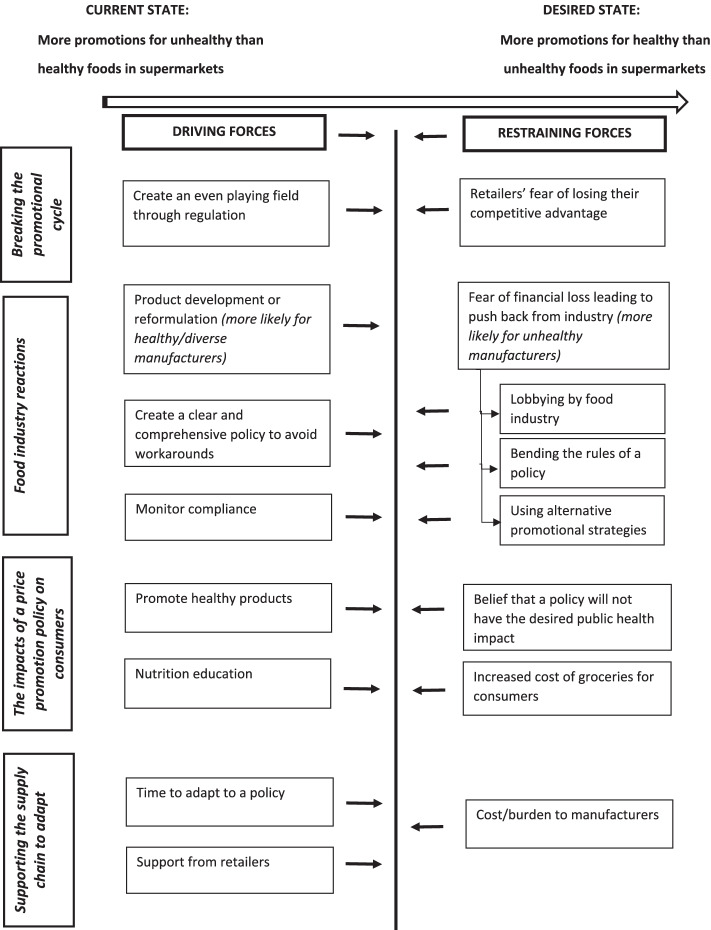
Force field model representing the driving and restraining forces to policy actions aimed at improving the healthiness of food and beverage price promotions in Australian supermarkets

Themes from the interviews are described below with illustrative quotes. A Force Field Analysis Model that represents the identified forces restraining or driving towards healthier price promotion practices in Australian supermarkets is depicted in Fig. 1.

### The process and considerations of setting price promotions

#### Price promotions are both planned and reactive

Participants described the typical process by which price promotions are set from the perspective of food retailers (supermarkets). They explained that promotions are managed at a category level (e.g., biscuits, soft drinks, dairy), overseen by category managers. Each category has its own sales targets and category managers compete to have price promotions in their category. Interviewees indicated that retailers use a promotional calendar to determine when and what products go on price promotion each week. At the start of the year, retailers populate the promotional calendar with high level themes, seasons, and key events throughout the year (e.g., Easter, Halloween). Around six months prior to the promotional period, decisions are made about what promotions will be run. Around three months before the promotion, details are confirmed e.g., the promotional price, what will be in the weekly promotion catalogue, and what the ‘hero product’ will be (e.g., the product featured on the front page of the catalogue and/or aisle end displays). Participants explained that ‘hero’ products typically need to have a deep discount (e.g., 50% off) to entice customers. At around 8 weeks prior to the promotional period, the promotions are locked into the calendar, with minimal opportunity for adjustments.

Participants indicated that, throughout the process of setting the promotional calendar, retailers communicate and negotiate with the suppliers (food manufacturers). Interviewees noted that suppliers typically have a promotional budget to spend on promoting their products in supermarkets. Price promotions are funded through supplier payments to the retailer, which can be in the form of a case deal (e.g., the supplier offers a discount off the wholesale price and recommends a maximum price at which the retailer should sell the product), a lump payment (e.g., for an off-location display or promotion on the front of the promotion catalogue), or a combination. Suppliers submit a proposal to the retailer mapping out the products and suggested price points they would like to have throughout the promotional calendar. The proposals for each category are then reviewed by the retailer, with amendments agreed through negotiation between suppliers and the retailer. The overwhelming view from interviewees, including those with experience working with retailers and manufacturers, was that retailers held more power than suppliers in the relationship when it came to setting promotions.

*“And obviously, you know, [the] Australian supermarket [sector] is … effectively a duopoly… the retailers hold all the power, and the retailers certainly have the ability to destroy manufacturers … and if you get in their bad books then … it can be hard to get back in.” (Participant 7, experience working with manufacturers and supermarkets).*

Interviewees indicated that when slotting in promotions, retailers will consider factors such as: when the product was last promoted, other activities in the category, and what other retailers typically do or are reported to be planning. Participants indicated that retailers closely monitor the promotions of other retailers to ensure they are offering prices and promotions that are competitive. Interviewees also explained that retailers also try to minimise promotions overlapping between different brands of similar products (e.g., two big soft drink companies). Likewise, suppliers will try to avoid promoting in different retailers at the same time.

Despite the promotional calendar being negotiated months in advance, interviewees indicated that, in some cases, price promotions were not planned well in advance or based on strategic insights. For example, interviewees indicated that unscheduled price promotions were sometimes implemented due to the pressure and incentives on retailer category managers to meet sales targets. Some interviewees described the retail environment as “frantic” and “reactive” when it came to meeting targets. Participants reported that managers closely monitor the sales performance of their category and if they are not on track to meet their volume target, they will often use price promotions as a reliable tool to increase sales. Sometimes this would mean that a retailer would put a popular brand on price promotion without the financial support of the supplier. In these instances, the retailer would expect to make a lower margin from selling the product at a discount in order to meet their volume target. Interviewees further indicated that product lines that have seen a reliable increase in sales from previous price promotions are often selected for repeated price promotions cycles.

*“… there’s such a reactive cycle in these sort of retailers [major Australian supermarket chains], like every day you get the [sales] results from the day before and every Monday morning you get the entire week before. Then, if it’s just a small drop [in sales] in a category or overall, people [staff at the retailer] panic a little and they know that the sort of go-to lever to sort of get it back on track is to go really hard on [heavily discount] a couple of promotions. So, … it’s sort of what people do just to fix the next week basically. And to break that cycle it’s just really hard.” (Participant 4, experience working with manufacturers and supermarkets).*

Participants identified that products were sometimes discounted to clear excess stock. Discounting products in this manner was not part of a strategic promotional plan but more of a practical issue at the store level.

“*Retailers also have a policy on how much stock they’re willing to have at any one time. The more stock they have on hand it means the more space they have to rent out for stock, so they want to keep as minimal stock back behind, you know, in storage. So they have tight policies as to how much stock they will take for how long.” (Participant 12, experience working with manufacturers)*

#### Price promotions drive sales and profit

For both retailer and supplier, profit was identified as a key motivator to setting price promotions. Consumers were regarded as highly price sensitive, making price promotions an effective promotional lever to drive sales and increase profit.

*“… it’s a competitive environment …, particularly Australia, … so, just in an objective sense, as a supermarket, you have to have the best deals. People are very price sensitive, much more than you might imagine they are… [Price promotions are] an important factor that drives their shopping - choice of shop, where are they going to shop, and what are they going to buy. So, it all comes back to competition and trying to get the biggest share of the market and trying to get people to buy more when they’re in the shop.” (Participant 1, experience working with manufacturers and supermarkets).*

According to those interviewed, retailers can profit from price promotions in multiple ways. Price promotions increase the volume of promoted items sold in store. Promotions can also drive customers into the store. Even if there is a low margin to be made from the discounted item, it is likely customers will purchase other full priced products whilst they are in the store. Additionally, it draws customers away from their competitors.

Suppliers also benefit from promotions by recruiting customers to their brand in a highly competitive environment, selling more volume of their products and improving economies of scale. Interviewees indicated that suppliers also consider how promotions can be used to maintain their positive relationships with the retailer. For example, some interviewees commented that even where price promotions proved costly to suppliers (e.g., through the financial contributions they make to retailers to secure the price promotion and/or the lower margin when the product is on promotion), if they were seen as beneficial to the retailer, they were considered worthwhile.

#### Health is not a consideration when setting price promotions

When asked about the healthiness of products on price promotion, interviewees noted that health was a very low priority when making decisions about price promotions.

*“Yeah, there’s a whole range of reasons [to put something on promotion] yeah and health and nutrition would not be part of that.” (Participant 12, experience working with manufacturers).*

For retailers, increasing revenue was perceived as the main reason for using price promotions, therefore retailers discount the products that sell best on promotion. According to those interviewed, unhealthy products typically met the criteria of selling better on promotion compared to healthy products. There were several reasons identified by interviewees as to why unhealthy foods sell better on promotion.

Firstly, interviewees indicated that products that are bought as impulse (unplanned) purchases sell in high volumes on price promotion. They explained that many ‘less healthy’ foods are impulse products, compared with healthier staple foods which are typically bought as part of a ‘planned shop’.

*“It’s not so much healthy or unhealthy. I think it’s more the impulse… no one goes into a shopping centre with a list of need more packets of M&Ms. That’s not on your list. But when you’re actually there and you see it’s half price, [they say to themselves], “like hey, why not? It’s delicious and I have a movie night coming or whatever.” So that’s – impulse categories are predominantly categories prone to promotional activities.” (Participant 5, experience working with manufacturers and supermarkets).*

Additionally, interviewees explained that ‘expandable’ categories (e.g., biscuits and crisps), where you can use more of the product or consume it faster once purchased, are more profitable to sell on promotion as the consumer is likely to purchase more of the product over time. Conversely, participants indicated that products that are shelf stable and likely to have a constant consumption rate (e.g., sugar and potatoes) are likely to be stored by the customer if purchased on price promotion, resulting in a financial saving but not an increase in overall product purchases over time. They indicated that a large proportion of unhealthy products were in expandable categories.

Some interviewees noted that, in many cases, ‘less healthy’ food suppliers are better able to finance promotions in store. These interviewees indicated that many of the largest food companies had ‘less healthy’ product portfolios and that their larger promotional budgets (compared to many smaller healthier food or beverage companies) facilitated greater use of price promotions.

*“I guess that these companies [large multinational companies with well-established global brands] have more marketing budget to then spend on these promotions…whereas, say, a small organic kombucha company… might not have a marketing budget” (Participant 8, experience working with supermarkets).*

Furthermore, healthier produce, such as fresh fruits and vegetables, are not typically branded, with multiple suppliers, making it more difficult for retailers to secure a supplier contribution to the promotion.

### The driving and restraining forces of shifting to healthier price promotions in supermarkets

#### Breaking the promotional cycle

Participants noted that a key motivation for retailers using price promotions was to gain a competitive advantage. Participants noted “fierce” competition between retailers, resulting in “pricing wars” whereby price promotions are repeatedly used as a promotional tool to draw customers into their store and away from the competition. Interviewees acknowledged that it would be difficult for a retailer to voluntarily change their price promotion practices through fear of losing customers to their competition. Furthermore, it was suggested that the competitive use of price promotions over a long period had led to price sensitive shoppers who expect and seek out promotions, thus making the pricing “battle” between retailers harder to break. The retailers’ reliance upon price promotion seemed to also be fuelled by the pressure to meet sales targets.

*“But [the retailers] can’t even like pull out of [the pricing wars]. They get so wedded into these price wars that neither one [of the two major supermarket retailers] wants to move first and pull away from that, because again it comes back to - they’re so scared of losing that competitive advantage and because people are very promiscuous in their shopping habits.” (Participant 1, experience working with manufacturers and supermarkets).*

In reflecting on the potential for government-led policy to reduce price promotions on unhealthy foods, multiple interviewees noted that for such a policy to work and disrupt the current promotional cycle, it would need to be fair and create an even playing field for retailers and suppliers. It was suggested that for an even playing field to be established, policies would likely need to be mandatory. Nevertheless, some participants reported that voluntary policy arrangements (e.g., guidelines endorsed by government for voluntary adoption by industry) would be favourable for industry. It was these interviewees’ perception that mandatory policies were suitable for issues relating to food safety where there is a high risk to consumers involved (e.g., allergy information), whereas voluntary initiatives were deemed more of a proportionate response to the health risks related to price promotions on unhealthy foods. Furthermore, some interviewees indicated that banning promotions on certain categories would likely put some companies out of business (e.g., companies that primarily sell foods categorised as unhealthy) making a voluntary approach more favourable to them. Critically, many interviewees noted that they did not believe that there would be widespread implementation of a voluntary policy on price promotions.

*“I mean, ideally you want it to be voluntary but that probably won’t work as well [as a mandatory policy], I guess. … I do think to get it to break those [price promotion] cycles and things that are going on at the moment, it probably needs to be mandatory in the beginning at least to sort of get the cycle broken and then turn around what is going on at the moment.” (Participant 4, experience working with manufacturers and supermarkets).*

#### Food industry reactions to policies to restrict price promotions on unhealthy foods

Interviewees noted that government policy to restrict price promotions on unhealthy food would not be perceived favourably by retailers or suppliers. Participants indicated that, in the first instance, there would be strong lobbying from the food industry to prevent the policy.

*“There’s going to be a massive push back [to any proposed policy action] where there’ll be a lot of … [food industry] heads coming together to go, how do we shut this up and make it go away.” (Participant 9, experience working with manufacturers and supermarkets).*

If mandatory restrictions on price promotions for unhealthy foods were implemented, participants thought that industry would find alternative ways to promote their products (e.g., lowering their permanent price, or promoting through placement strategies) or bend the rules (e.g., finding ‘grey areas’ in the definition of what is healthy and unhealthy) to ensure sales volumes do not drop. To prevent ‘work-arounds’, interviewees noted that compliance to the policy would need to be closely monitored. It was also felt that any policy would need to be very clear and comprehensive, this included clearly defining healthy and unhealthy, including broader in-store promotional activity, and considering other aspects of the food environment (e.g., fast food outlets).

*“Some of the products in that [biscuits] category are healthier and some of them are quite obviously not healthy. They would be the kind of categories that they [food companies] would be starting to play around in. “What if I put a brown wheat biscuit on promotion rather than a chocolate biscuit?” … “What if I put the brown wheat biscuit next to the chocolate biscuit and put the brown wheat product on promotion”, would people then go “oh, that chocolate biscuit looks nice, I’ll get that”. I think they would have to get – they would play within the rules. … [but] they would come so close to breaking the rules that it would … be difficult to police I think. I think they would be – they’d be hiring teams of consultants to work out, “Right, we’re in this spot, the landscape has changed, how can we make the best out of this poor situation?” (Participant 7, experience working with manufacturers and supermarkets).*

Reformulation or product development was another suggested reaction food manufacturers may take in response to a policy restricting price promotions on unhealthy foods. Participants commented that the likelihood of a manufacturer opting to reformulate or shift their focus to healthier products was moderated by the adaptability of the company and the healthiness of their current product portfolio: companies with primarily unhealthy portfolios would likely find it more difficult to adapt compared to companies that sold healthier products or a range of products.

#### The impact of a price promotion policy on consumers

There were differences in views amongst interviewees regarding the likely public health gains from a reduction in price promotions on unhealthy foods. Some participants believed that it would not change diet quality overall because people would switch to cheaper options within particular unhealthy product categories. These participants indicated that changes to price promotions were deemed unlikely to be enough to change unhealthy purchasing behaviour as there were other factors perceived to contribute to unhealthy choices, such as convenience, brand loyalty, habits, food preferences, cooking skills and nutritional knowledge. However, some felt that price promotions encourage increased purchasing and, therefore, a policy to change the nature of price promotions would be effective in changing purchase behaviour, particularly if healthier options were promoted.

* “I wonder if [a successful policy could be] less about disallowing promotions on unhealthy things but forcing a certain allocation or proportion of promotions to things that are healthy so that every week… there might be something that said: of the food products [promoted] in that range, at least half of them have to be healthy by some measure, or something like that.” (Participant 8, experience working with supermarkets).*

There were also opposing views regarding the likely financial impact to consumers of a policy restricting price promotions on unhealthy foods. Some participants suggested that by reducing or removing price promotions the average price of a grocery shop (typical basket of goods) would increase overall. Additionally, they felt that the removal of price promotions may result in retailers increasing the prices of other products to compensate for the loss in profit previously made through price promotions. Furthermore, it was thought that the policy may disadvantage certain shoppers who will continue to buy unhealthy foods when at a regular price and may forgo healthier items on their shopping list to keep the cost of their basket down.

However, some interviewees indicated that retailers may lower their permanent price points if they were unable to practice high-low pricing strategies (a common pricing strategy where products are sold at a relatively high price and then sold at a lower price during a price promotion period that is frequently repeated). Participants noted that with the current high-low pricing strategy, many products e.g., impulse products such as confectionery and sugary drinks, sell poorly when at the regular (high) shelf price. The greatest proportion of sales for these products come from when the product is discounted in price. Therefore, if price promotions were restricted in these categories, it was hypothesised that retailers would reduce the permanent price to somewhere midway between the low and high price to attract customers.

It was suggested that focusing on increasing the number of price promotions on healthy foods as opposed to removing price promotions on unhealthy products might be more acceptable to consumers. It was also the opinion of participants that nutritional education and consumer buy-in would facilitate healthy choices.

*“I think if you want people to change their eating behaviours there are better ways to do it [than eliminating price promotions on unhealthy foods], and that requires … investment in education. … I just feel that more can be done in terms of public consumer education in increasing people’s knowledge, and with knowledge people can then make better choices and they can determine what they want to choose, when they want to eat and how much they want to eat it.” (Participant 12, experience working with manufacturers).*

#### Supporting the supply chain to adapt

Interviewees highlighted that the cost and burden of changing promotional practices was likely to prove a barrier to change for suppliers. For example, interviewees cited the costs involved in reformulation, changing equipment, and potential job losses. On the other hand, it was suggested that a policy to reduce the number of price promotions on unhealthy foods may result in less work for the retailers as there may be less planning and in-store work to set up promotions, although the impact would depend on whether the overall number of price promotions each week changed.

Participants identified several driving forces that could facilitate implementation of a policy to reduce price promotions on unhealthy foods. Some participants proposed incentivising companies to shift towards manufacturing healthier products or subsidising the promotion of healthier products. For example, it was suggested that the government or retailers could provide financial support to promote healthy foods. One participant suggested subsidising promotions for healthy foods from smaller suppliers, thereby allowing smaller suppliers to grow and incentivising the development of healthy foods.

Participants also recommended that there be sufficient time for the industry to adapt (e.g., reformulate, put new practices in place) before a policy was implemented. Several participants suggested a phased-in approach such that a target would be set on the proportion of promotions that are healthy and gradually increase that proportion over time, whilst simultaneously reducing the overall number of promotions.

*“And it [a policy restricting price promotions on unhealthy foods] needs to be sign-posted a long way in advance… I think yeah it needs [to be announced] a good two or three years [in advance] … to allow those manufacturers and those retailers to, you know, change and adapt, set the different ground rules, reformulate if need be.” (Participant 11, experience working with manufacturers)*

Lastly, several participants considered support from retailers to be an important facilitator to policy implementation. As retailers were viewed as having a large degree of control in setting price promotions, it was believed that they could act as an important gatekeeper to enforce a policy as well as reinforce corresponding healthy eating messages to shoppers.

## Discussion

In this study, twelve interviews were conducted with food and beverage industry stakeholders to understand the process by which price promotions are set, and the acceptability and feasibility of implementing policy actions that modify the healthiness of food and beverage price promotions in Australian supermarkets.

From the interviews, it was identified that current price promotions are highly lucrative for retailers and their suppliers. Therefore, a policy that restricts price promotions for unhealthy foods would likely be strongly opposed by many food industry stakeholders. This is consistent with industry responses observed to other policies designed to improve population diets. For example, industry opposition to the original price promotion consultation in the UK (2018) [43] may have contributed to the weakening of the policy design. The initial proposal of a restriction on all unhealthy food and beverage price promotions was watered down to a policy that restricts only on volume-based price promotions (e.g., two-for-one), meaning temporary price reductions are still allowed under the proposed law [19]. Lobbying from the food industry has been identified as a key barrier to implementing healthy eating policies, leading organisations such as the WHO to advocate for nutrition policy to be developed by governments and to be safeguarded against influences from the food industry [44].

Interviewees agreed that if a mandatory policy to reduce price promotions on unhealthy foods was implemented, industry would likely promote their unhealthy products in alternate ways (e.g., lowering their permanent price, or promoting through placement strategies). Interviewees also identified the importance of having clear definitions of healthy and unhealthy foods, and monitoring compliance. Industry responses to the UK consultation on restricting promotions revealed a similar sentiment, requesting clear and detailed guidance on the scope of the restrictions and the enforcement regime from the government. This is echoed by academics who have recommended that industry initiatives require clear targets for implementation; objective and transparent monitoring and evaluation; and meaningful sanctions for non-compliance [45–47]. Alternate promotion and/or pricing strategies and the implication on the healthiness of foods sold at supermarkets should be closely monitored when the UK policy comes into force.

In considering the potential impact of policies to restrict price promotions on unhealthy foods, the study indicated that policy action may incentivise increased product development or reformulation in favour of healthy products. Giving suppliers time to adapt and/or phasing in the regulation with stepped targets may facilitate such reformulation or product development. Reformulation following mandatory nutrition-related policies has been seen elsewhere, for example, an evaluation of the UK sugar-sweetened beverage tax found that the tax resulted in manufacturers reformulating products which substantially reduced the sugar content [48]. More generally, many large food manufacturers operating in Australia already have diverse product portfolios, consisting of a range of both healthy and unhealthy products [49]. In many cases, these manufacturers would likely have multiple opportunities to shift towards adopting price promotions on their healthier products within each category. However, their willingness to do that, the likely response from supermarkets, and the corresponding impact on population diets need to be explored in more detail.

This study indicated that a policy to restrict price promotions on unhealthy foods was likely to be most effective if it was mandatory, with voluntary action unlikely to be widely adopted due to the current “promotional cycle” and competitive dynamics. Similarly, responses from the consultation process in the UK’s proposal to ban promotions revealed that a voluntary initiative was unlikely to work as there would need to be a level playing field across the retail sector [22]. The likely benefit of mandated public health policies in the area of nutrition is evident from previous voluntary initiatives that have fallen short of their public health objectives [50]. For example, a review of the Health Star Rating (HSR), a voluntary front-of-pack labelling scheme for packaged foods used in Australia, found that although there has been substantial uptake of the policy by some manufacturers, the overall uptake is still considered to be sub-optimal (only around 40% of eligible products had a HSR, most of which were on products with higher i.e., healthier, HSRs) [51, 52].

While some industry representatives included in this study indicated that restrictions of price promotions on unhealthy foods were likely to be effective, other participants indicated that policy action in this area was unlikely to curb consumer demand for unhealthy foods and stressed a preference for consumer education instead. This view is comparable to previous research exploring retailers’ perspectives of healthy eating initiatives, where the primary reason for stocking and/or promoting unhealthier foods is cited as customer demand [28–31]. Food industry arguments about the need for consumer education (rather than industry regulation) to address unhealthy diets have frequently been identified [53–55]. A strong body of evidence has noted that this narrative is designed to deflect the need for industry action to improve population health and maximise industry profits (often at the expense of public health) [56–59]. Furthermore, education interventions alone have been shown to be ineffective without greater action to alter food environments to support healthier choices [12, 13, 60]. Interestingly, a recent shopper survey from the Republic of Ireland found that over 90% of participants wanted to see more promotions on fruits and vegetables and the majority wanted less on products such as cakes, biscuits, soft drinks, and confectionery [27]. Additional research is needed to understand the effects of altering the proportion of unhealthy to healthy price promotions on purchase behaviour and consumer views on such policies.

### Strengths and limitations

To our knowledge, outside of information gleaned from the recent UK government consultation on price promotion policy, this is the first study to explore both food retailers’ and suppliers’ reflections on policy actions that target price promotions to encourage healthy dietary behaviours. Furthermore, this study included participants with substantial expertise in the food industry, providing rich, in-depth data on food and beverage price promotions in Australia.

However, there were several limitations to this study. Firstly, a number of current food industry employees in Australia declined participation in the study, and the included participants were primarily identified through LinkedIn, potentially biasing the sample toward those active on this social media platform and ex-employees of food companies. Nevertheless, the included participants had extensive experience in the food industry and were able to offer deep insights into food and beverage price promotion strategies in Australian supermarkets, both from retailer and supplier perspectives. Secondly, the personal experiences and beliefs of the researcher (a public health PhD student) will have influenced the interpretations of the findings. To reduce potential bias from the researcher’s own experiences and assumptions, reflexivity was practiced throughout the data collection and analysis. Additionally, themes and a transcript were cross checked by another researcher.

Lastly, this study was only conducted with industry stakeholders based in Australia. The supermarket landscape and marketing tactics in Australia are context specific and are likely to differ across countries. For example, Australia is dominated by two main supermarket retailers [61], Australians have been found to be some of the most price sensitive grocery shoppers [62], and the proportion of grocery sales purchased while on price promotions in Australia is one of the highest in the world [63]. It is likely that these contextual differences will be reflected in the barriers and enablers to a policy on price promotions observed in this study. While the use of well-established theories of organisational change in analysing the study results is likely to increase the generalisability of the findings (especially in other high-income countries with similar regulatory systems to Australia), relevant stakeholder perspectives from other countries and contexts should be explored.

### Implications for public health policy

Unhealthy diets are a leading contributor to ill health in Australia and globally [64]. Accordingly, governments are actively seeking strategies to improve population diets [6, 65, 66]. As price promotions on unhealthy foods and beverages are likely a contributing factor to unhealthy diets, governments need to actively consider ways to address their impact as part of a comprehensive approach.

This study, along with other research, suggests that for a policy to reduce price promotions on unhealthy foods to be successful, the policy would need to be comprehensive (clearly defining healthy and unhealthy products, applying to a range of settings, and including broader in-store promotional activities such as placement strategies); be mandatory; include extensive compliance monitoring (including the monitoring of broader marketing tactics to ensure there is not an increase in compensatory marketing strategies); incentivise the promotion of healthy products; and consider phasing in targets to shift the proportion of healthy and unhealthy price promotions. Moreover, to counter likely industry responses, it would be critical that policy action to restrict price promotions be embedded within a comprehensive nutrition policy (including taxes, consumer education, advertising and online marketing restrictions, etc.) targeting multiple marketing strategies and techniques. The findings from this study strongly imply that industry will resist a policy that limits price promotions and provides insights into the arguments they will use to defend their position. To counter these industry arguments, it is important for researchers and public health advocates to generate evidence on the impacts a policy to restrict price promotions on unhealthy foods will have on purchasing behaviour, diet quality, financial cost to the public, and the public’s perception of a policy.

## Conclusions

In this study, interviews with food and beverage industry stakeholders revealed that the industry would likely oppose government action to reduce the number of price promotions on unhealthy foods and beverages due: to fear of losing competitive advantage; potential financial loss for food retailers and their suppliers; a perception that restrictions on price promotions for unhealthy products will not impact health; and a perception that such action would result in increased financial cost to consumers. Nevertheless, the study found that the following forces would drive implementation: mandatory regulation; extensive compliance monitoring; support for promoting healthy products; consumer education; and sufficient lead time and support from retailers for implementation. The findings indicate that policy actions designed to improve the healthiness of price promotions would likely need to be mandatory in order to prove effective. Furthermore, to counteract likely industry responses to policy implementation (e.g., moves to other marketing techniques), policies to restrict price promotions for unhealthy foods and beverages would likely be most effective when implemented as part of a broader multi-pronged policy to restrict the marketing of unhealthy foods and improve population diets. The insights from this study will be useful to policy makers to understand the key industry considerations when designing an appropriate policy response to unhealthy diets in Australia

## Supplementary Information

**Additional file 1.**

## Data Availability

The data used during the current study are available from the corresponding author on reasonable request.
