# Supplementary material for: Food industry perspectives on potential policies targeting unhealthy food and beverage price promotions in Australian supermarkets
Source: BMC Public Health. 2022 Jul 26;22:1423. doi: 10.1186/s12889-022-13812-7 (PMC9322738; doi:10.1186/s12889-022-13812-7)
Supplement: Supplementary file 1 — Additional file 1. [file 12889_2022_13812_MOESM1_ESM.docx]

**Appendix**

Interview guide used in interviews with food and beverage professionals to explore price promotion policies and practices in the Australian supermarket setting.

| **Objective** | **Key Questions** |
| --- | --- |
| To understand the current process for setting and implementing food/beverage price promotions in Australia | - What is your understanding of how food and beverage price promotions are set? - Who are the key stakeholders involved in deciding price promotions? - What are the key considerations for retailers/food suppliers/manufacturers when setting price promotions? - How much consideration is given to healthy eating or public health when making decisions about price promotions? - Currently, price promotions in Australian supermarkets favour less healthy products as opposed to healthier products, what might the reasons for this be? |
| To explore the acceptability of policy actions that modify food and beverage price promotions to encourage healthy eating, and to understand facilitators to acceptability | - What are your thoughts on potential policies to reduce the purchase of unhealthy products on price promotions in Australia? - What might policies to reduce the purchase of unhealthy products on price promotions in Australia need to look like? - What would it take to get buy-in from stakeholders? - Are there any other strategies you can think of where price promotion practices could be more in line with health? |
| To explore the feasibility of implementing policy actions that reduce price promotions on unhealthy food and beverages, and to understand barriers and facilitators to implementing such a policy | - Thinking about these strategies to reduce price promotions on unhealthy foods, how do you think that would work practically for your organisation? - What are the potential barriers to reducing price promotions on unhealthy foods for organisations such as yours? - What would it take for that not to be a barrier? |
